# Short-Term Regulation of Murine Colonic NBCe1-B (Electrogenic Na^+^/HCO_3_^−^ Cotransporter) Membrane Expression and Activity by Protein Kinase C

**DOI:** 10.1371/journal.pone.0092275

**Published:** 2014-03-18

**Authors:** Oliver May, Haoyang Yu, Brigitte Riederer, Michael P. Manns, Ursula Seidler, Oliver Bachmann

**Affiliations:** Department of Gastroenterology, Hepatology and Endocrinology, Hannover Medical School, Hannover, Germany; Tohoku University, Japan

## Abstract

The colonic mucosa actively secretes HCO_3_^−^, and several lines of evidence point to an important role of Na^+^/HCO_3_^−^ cotransport (NBC) as a basolateral HCO_3_^−^ import pathway. We could recently demonstrate that the predominant NBC isoform in murine colonic crypts is electrogenic NBCe1-B, and that secretagogues cause NBCe1 exocytosis, which likely represents a component of NBC activation. Since protein kinase C (PKC) plays a key role in the regulation of ion transport by trafficking events, we asked whether it is also involved in the observed NBC activity increase. Crypts were isolated from murine proximal colon to assess PKC activation as well as NBC function and membrane abundance using fluorometric pH_i_ measurements and cell surface biotinylation, respectively. PKC isoform translocation and phosphorylation occurred in response to PMA-, as well as secretagogue stimulation. The conventional and novel PKC inhibitors Gö6976 or Gö6850 did not alter NBC function or surface expression by themselves, but stimulation with forskolin (10^−5^ M) or carbachol (10^−4^ M) in their presence led to a significant decrease in NBC-mediated proton flux, and biotinylated NBCe1. Our data thus indicate that secretagogues lead to PKC translocation and phosphorylation in murine colonic crypts, and that PKC is necessary for the increase in NBC transport rate and membrane abundance caused by cholinergic and cAMP-dependent stimuli.

## Introduction

Electrogenic Na^+^/HCO_3_^−^ cotransporter NBCe1 exists in 2 basolaterally localized variants (SLC4 family members NBCe1-A and NBCe1-B) with major differences in their transport direction, stoichiometry, expression pattern, and regulation [1]. NBCe1-B is more widely distributed, and is thought to act as a base-loading mechanism in the gastrointestinal epithelium together with NBCn1 to enable cellular pH regulation and transepithelial HCO_3_^−^ transport [2]–[4]. NBCe1-A, on the other hand, is primarily found in the kidney, where it mediates HCO_3_^−^ reabsorption in concert with apical Na^+^/H^+^ exchanger NHE3 [1].

Early on, the importance of physiologically relevant regulatory pathways has been investigated, and these studies have revealed a differential regulation of NBCe1-A and NBCe1-B by cholinergic and cAMP-dependent stimulation [2], [5]–[8]. These differences have been attributed to the regulatory properties of the respective variant arising from the primary structure [9]–[11], but also to cell-type specific factors [5], [10], underlining the importance of data derived from experiments using native tissue rather than heterologous expression systems.

Protein kinase C (PKC) has been shown to modulate HCO_3_^−^ transport in various experimental systems [12]–[18]. The described short-term functions of PKC are complex and isoform-specific and involve direct effects on transporters and channels [13], [19], the modulation of other signal transduction pathways [15], [16], and an influence on transporter/channel trafficking [20], [21]. Although basolateral HCO_3_^−^ uptake in the intestinal epithelium by specific transporters is believed to be essential for intracellular pH regulation and further functions of HCO_3_^−^, and also rate-limiting for transepithelial HCO_3_^−^ transport, the role of PKC in the regulation of intestinal HCO_3_^−^ uptake has not been studied in detail.

In renal cells, early functional data indicates a stimulatory effect of PKC on Na^+^/HCO_3_^−^ cotransport: In cultured proximal tubule cells pre-treated with ethylisopropyl amiloride, ^22^Na uptake is significantly enhanced in the presence of phorbol ester and HCO_3_^−^ [22]. Similarly, fluorometrically measured Na^+^/HCO_3_^−^ cotransporter activity was stimulated by PMA in isolated proximal tubules [23]. The question thus arises whether PKC also activates NBC in the gut. It has to be kept in mind, however, that Na^+^/HCO_3_^−^ cotransporter regulation displays fundamental differences between the kidney and the gastrointestinal tract for other pathways, making the results from renal cells not readily transferable to the gut epithelium. In the latter, the relevance of PKC in regulating basolateral base-loading transporters like NBC is largely unknown.

We could previously demonstrate that cholinergic stimulation of NBC in isolated murine colonic crypts is partially reversible by treatment with PKC inhibitors [8]. However, it is not clear whether PKC is involved in the cAMP pathway, which differentially regulates NBCe1 [2], [6], and/or in subcellular redistribution [24], [25] of the transporter in the intestine. We therefore set off to study the regulation of NBCe1 by protein kinase C in native colonic tissue.

## Materials and Methods

### Materials

The polyclonal anti-NBCe1 antibody K1A directed against the cytoplasmic COOH-terminus common to the NBCe1-A and NBCe1-B subtypes was generously supplied by Walter Boron [24], [26]. The anti-PKC-α,-δ and-ε antibodies, the corresponding blocking peptides as well as the anti-phospho-PKC antibodies were from Santa Cruz Biotechnology (Santa Cruz, CA, USA). 12-(2-cyanoethyl)-6,7,12,13-tetrahydro-13-methyl-5-oxo-5H-indolo (2,3-a) pyrrolo (3,4-c)-carbazole (Gö-6976), and 2-[1-(3-dimethylaminopropyl)-1H-indol-3-yl]-3-(1H-indol-3-yl)-maleimide (Gö-6850; bisindolylmaleimide I) were from Merck (Darmstadt, Germany). Alexa Fluor 488 goat anti-rabbit IgG, Alexa Fluor 488 goat anti-mouse IgG, Nigericin, and 2′,7′-biscarboxyethyl-5(6)-carboxyfluorescein (BCECF/AM) were all from Invitrogen (Life Technologies, Darmstadt, Germany), and the anti-β-actin-antibody was from Abcam (Cambridge, UK). Forskolin, carbachol, and phorbol-12-myristate-13-acetate (PMA) were from Sigma (Taufkirchen, Germany). Sulfosuccinimidyl-2-(biotinamido)ethyl-1,3-dithiopropionate (Sulfo-NHS-SS-Biotin) was purchased from Pierce Biotechnology, Rockford, IL, USA. All other chemicals were either obtained from Sigma or from Merck at the highest grade available.

### Animals

C57BL/6 mice were kept in the animal facility of Hannover Medical School under standardized light and climate conditions, had access to water and chow *ad libitum*, and were used for the experiments at the age of 3 months. All experiments followed approved protocols and guidelines from the Medical School of Hannover (Permit Number: AZ 2012/10) and the local authorities for the regulation of animal welfare (Niedersächsisches Landesministerium für Verbraucherschutz und Lebensmittelsicherheit).

### Preparation of colonic crypts

After CO_2_ narcosis, mice were sacrificed by cervical dislocation (in accordance with the German law for animal protection [TierSchG §4 Abs. 1]), keeping CO_2_ exposure as short as possible to minimize the effect on acid/base transporters. Subsequently, crypts were isolated as previously described [24] from a 3–4 cm proximal colonic segment filled with and incubated in EDTA-containing buffer (in mM: 127 NaCl, 5 KCl, 1 MgCl_2_, 5 sodium pyruvate, 10 HEPES, 5 EDTA, 1% BSA, and 5 glucose, pH 7.4, gassed with 100% O_2_) at 37°C for 12 min, harvested after gentle agitation, immersed in ice cold buffer A and stored on ice until use.

### Microfluorometry

Intracellular pH (pH_i_) was measured in BCECF-loaded crypts using a video imaging system exactly as previously described [8], [24]. After intracellular acidification with an NH_4_^+^-prepulse protocol by subsequent perfusion with buffers C, D and B (see Table S1), pH_i_ recovery rates were measured in the presence of the intended compounds and 700 μM dimethylamiloride (DMA), which we have shown to entirely inhibit Na^+^/H^+^ exchange activity in murine colonic crypts. Calibration of the 440- to 490-nm ratio was performed using the high K^+^-nigericin method [8].

### Cell surface biotinylation

Isolated murine colonic crypts were immersed in buffer B (37°C, pH 7.4) and treated with PMA, secretagogues and/or PKC inhibitors according to the experimental protocol, incubated twice with biotinylation buffer (in mM: 154 NaCl, 10 borate, 7.2 KCl, and 1.8 CaCl, pH 9.0, 1 mg/ml Sulfo-NHS-SS-Biotin), washed and lysed as previously described [24]. Biotinylated protein was retrieved using streptavidin beads (NeutrAvidin, Thermo Fisher Scientific, Rockford, IL, USA).

### Immunoblotting

A previously validated amount of each fraction yielding signals within a linear range of optical density suitable for semi-quantification was loaded, and size fractionated as described by Hillesheim et al. [27]. Western blots were loaded with anti-NBCe1 antibody (1∶500 in TBS-Tween) or anti-β-actin-antibody (1∶2500 in TBS-Tween) and incubated overnight at 4°C. The secondary antibody (goat anti-rabbit IgG conjugated to horseradish peroxidase, KPL, Gaithersburg, MD, USA) was diluted (1∶10.000 for anti-NBCe1) in TBS-Tween and incubated for 1 h at room temperature. The antigen-antibody complexes on the membranes were visualized with a Western blotting detection reagent (Amersham Pharmacia Biotech), and the image was captured on Hyperfilm (Amersham Biosciences, GE Healthcare, München, Germany). ODI (optical density integrated) was determined as described [24], and normalized to the internal β-actin control.

### Immunohistochemistry

To estimate the distribution of PKC isoforms in relation to NBCe1, the mid colon of C57B/6 mice was prepared. NBCe1 was visualized on cryosections stained with the K1A-antibody exactly as previously described [24]. For the PKC isoforms, mid-colon preparations were embedded in paraffin, sectioned with a Microm HM335E microtome (Microm, Walldorf, Germany) at 2 μm thickness, and deparaffinized. For improved antibody binding, slides were boiled in DAKO Target Retrieval Solution at pH 9 at 96°C for 20 min. After a 6 h blocking period with 10% goat serum, slides were incubated for 24 h at 4°C with the anti-PKC (α,δ,ε) antibodies (1∶100) in antibody diluent (5% goat serum in PBST), followed by the secondary antibodies (Alexa Fluor 488 goat anti-rabbit IgG or Alexa Fluor 488 Donkey anti-mouse IgG 1∶1000, 1 h at room temperature). The slides were then washed four times and embedded in DAKO fluorescent mounting medium. Images were acquired using an Olympus BX 60 microscope (camera: Olympus CX 50) and processed with ImageJ (NIH, Bethesda, MD; http://rsb.info.nih.gov/ij/).

### Statistical analysis

Results are given as means ± SE. Proton fluxes were calculated by multiplying the initial steep pH_i_ slope after the re-addition of Na^+^, which was determined by regression analysis, with the total buffering capacity at the initial pH_i_, including the intrinsic buffering capacity (β_i_) and, in addition, the CO_2_-dependent buffering capacity for CO_2_/HCO_3_^−^-containing solutions. Student's t-test in its paired and unpaired form, where appropriate, was used for pair-wise tests, and ANOVA was used for multiple comparisons (ANOVA for correlated samples in the biotinylation experiments), and Tukey's HSD (Honestly Significant Difference) Test as a post-hoc test. A p-value less than 0.05 was considered statistically significant.

## Results

**Figure 1:**
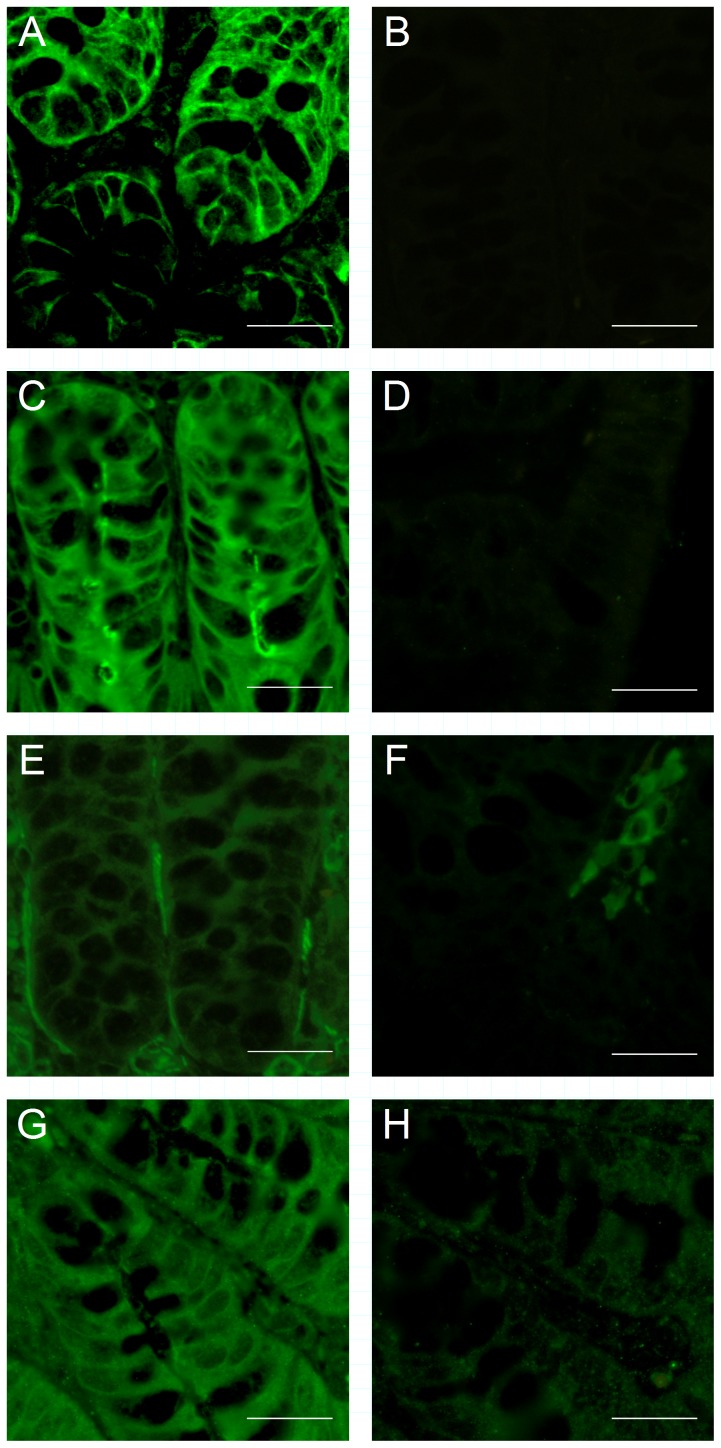
Immunohistochemical staining of NBCe1 and PKC isoforms in murine colonic tissue sections. NBCe1 (A) is expressed in the basolateral membrane of colonic crypts. Cytoplasmatic expression was observed for PKCα (C), δ (E) and ε (G) isoforms, with an apparent slight accumulation of the signal in the vicinity of the cell membrane for PKC α and PKC ε. Control experiments without the secondary antibody (NBCe1, B) and blocking peptides (PKC isoforms, D/F/H) showed no specific signal. Size of the scale bar is 50 μm.

In this study designed to investigate the role of PKC during secretagogue-associated activation of Na^+^/HCO_3_^−^ cotransporter (NBC), we first asked how PKC isoforms are subcellularly localized in murine colon, and whether PKC activation by forskolin and carbachol occurs in isolated crypts. To this end, we stained murine colonic sections with antibodies against NBCe1 as well as conventional and novel PKC isoforms (α, δ, ε). As observed previously, NBCe1 was found in the basolateral membrane, with some signal in the cytoplasm likely representing inactive NBCe1 in the resting state [24]. All PKC isoforms were expressed in the cytoplasm of the epithelial cells, with a somewhat stronger expression in surface cells, and an accentuation of PKC -α and -ε expression in the vicinity of the apical membrane (Figure 1). Since all PKC isoforms tested were present in crypt cell cytoplasm, an interaction with NBC, which is expressed in the basolateral crypt membrane [2], [24], is theoretically conceivable.

**Figure 2:**
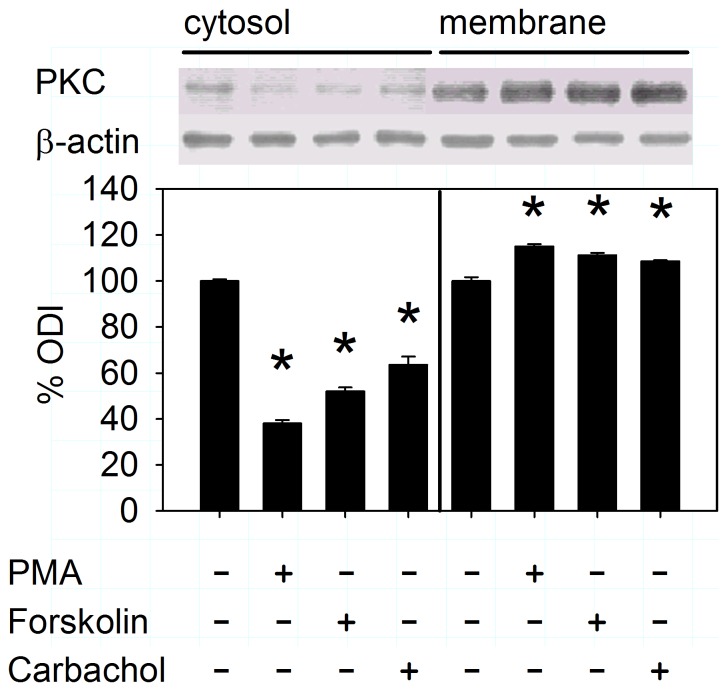
PKC translocation in response to PMA and secretagogues. PKC membrane expression as assessed by surface biotinylation and probing with a general PKC antibody significantly increased after exposure to PMA (100 nM), forskolin (10^−5^ M), or carbachol (10^−4^ M, 10 min each), which was paralleled by a decrease in cytosolic PKC expression [ODI: optical density integrated; *:p<0.05 vs. unstimulated, n =  6 preparations from separate mice in each group, ANOVA for correlated samples followed by Tukey's HSD (Tukey's honestly significant difference test), values expressed as % of the unstimulated control for cytosol and membrane, respectively].

**Figure 3:**
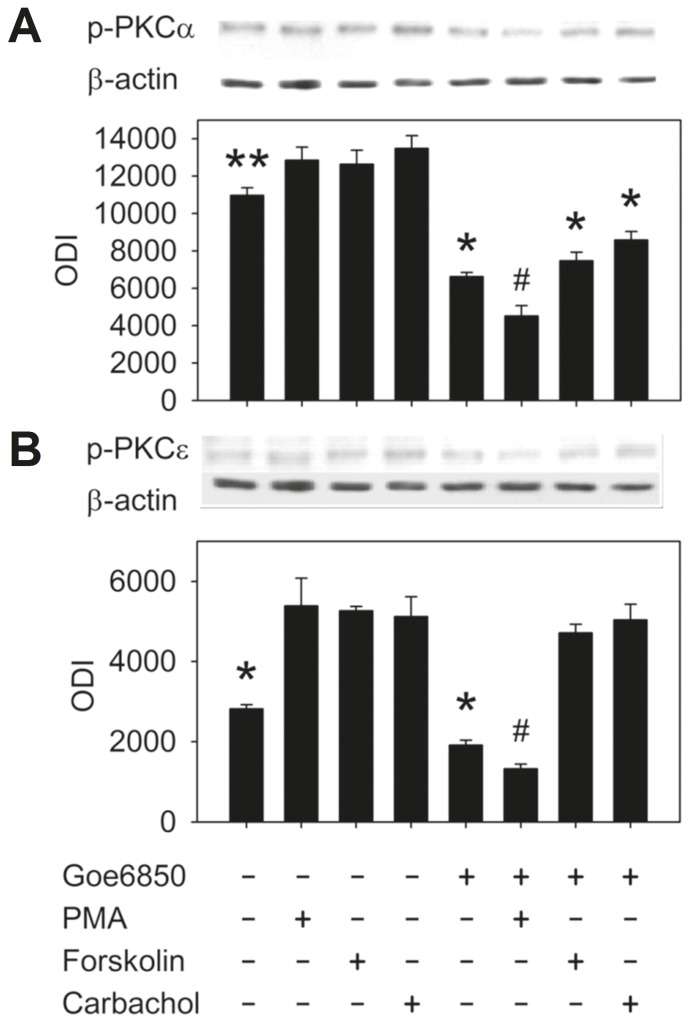
PKC phosphorylation in response to PMA and secretagogues. Phosphorylation was assessed in crypt lysates using the respective antibody recognizing the phosphorylated isoform. All compounds caused an increased in phosphorylated PKC-α (A) and PKC-ε (B; preincubation 10 min. 37°C; **:p<0.05, *:p<0.05 vs. unstimulated). When Gö6850 (5 μM) was added 10 min prior to stimulation, the effect of stimulation on p-PKC-α was completely reversed (A; #:p<0.01, *:p<0.05). PKC-ε phosphorylation, however, was only inhibited in the case of PMA (100 nM), but not secretagogues (B; #:p<0.01, *:p<0.05; n =  6 preparations from separate mice in each group, ANOVA for correlated samples followed by Tukey's HSD).

PKC activation occurs in parallel with its translocation to the membrane, and we thus performed experiments to verify whether this can be observed in our model. In a first approach, cell surface biotinylation with a general PKC antibody was carried out (Figure 2). In a second set of experiments, the effect on PKC-α and PKC-ε phosphorylation was studied using an antibody directed against the phosphorylated PKC isoforms (Figure 3). As expected, direct PKC activation by PMA induced PKC translocation to the membrane, as well as increased PKC phosphorylation. Interestingly, however, the secretagogues forskolin and carbachol also led to an increase in PKC translocation and phosphorylation. PKC-α phosphorylation by secretagogues was completely reversed by Gö6850 (bisindolylmaleimide I), while for PKC-ε, only PMA-induced PKC phosphorylation, but not the secretagogue effect was significantly diminished. This may indicate a differential function of these conventional and novel PKC isoenzymes in secretagogue vs. phorbol ester dependent stimulation.

**Figure 4:**
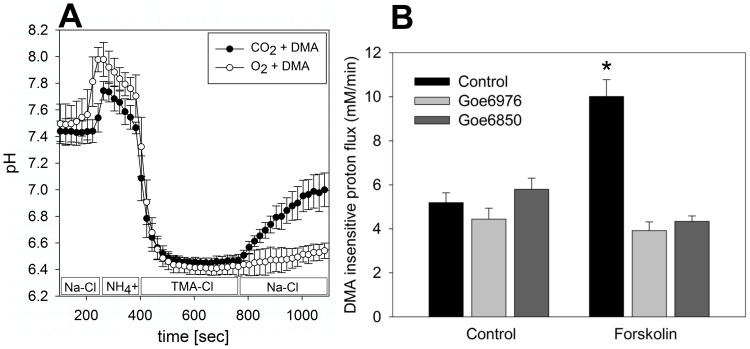
pH-microfluorometrical determination of NBC activity in the presence of PKC inhibitors with and without forskolin stimulation. NBC transport rates were determined as the Na^+^- and CO_2_/HCO_3_^−^-dependent, DMA-insensitive proton flux rates during pH_i_ recovery from an acid load. A: Average pH_i_ trace (n = 5) illustrating the pH_i_ recovery protocol, where crypts were acidified with an NH_4_ “prepulse” to a pH of 6.4±0.2 in Na^+^-free buffer (TMA-Cl) and let to recover after Na^+^ re-addition in the presence of 700 μM DMA to block all Na^+^/H^+^ exchanger isoforms. In the absence of CO_2_/HCO_3_^−^, no significant pH_i_ recovery was observed, but in its presence, there was a steady pH_i_ increase representing Na^+^/HCO_3_^−^ cotransport (NBC; [2], [8]). B: Left side: Incubation with Gö6976 (5 μM, 10 min prior to stimulation, light grey bars) or Gö6850 (5 μM, 10 min prior to stimulation, dark grey bars) did not alter the control proton flux rates (solid bars; p = n.s.). Right side: Stimulation with forskolin led to the previously observed significant stimulation of NBC (10.2±0.8 vs. 5.3±0.4 mM/min; *:p<0.05). Preincubation with either of the PKC inhibitors completely reversed this effect (n = 5–7 experiments from separate mice, p = n.s., ANOVA for independent samples followed by Tukey's HSD).

Next, we sought to investigate the role of PKC during secretagogue-dependent NBC regulation in colonic crypts. We had previously shown that carbachol-induced NBC activation is abolished by Gö6850 and Gö6976, with a non-significant trend towards a weaker effect of Gö6976 [8], which only inhibits conventional PKC isoforms [28]. Following up on these data, we fluorometrically measured NBC activity with and without forskolin stimulation and PKC inhibitors, respectively. Na^+^/HCO_3_^−^ cotransporter activity was fluorometrically determined in isolated murine colonic crypts [2], [8]. Na^+^/H^+^ exchange (NHE) was pharmacologically separated using a dose of di-methyl-amiloride which is inhibitory for all NHE isoforms present in the colon [8]. Similar to carbachol, Gö6850 and Gö6976 both completely reversed the stimulatory effect of forskolin, indication that PKC is involved in PKC stimulation by both secretagogues (Figure 4). There was trend towards lower flux values during stimulation with forskolin in the presence of PKC inhibitor vs. inhibitor alone.

**Figure 5:**
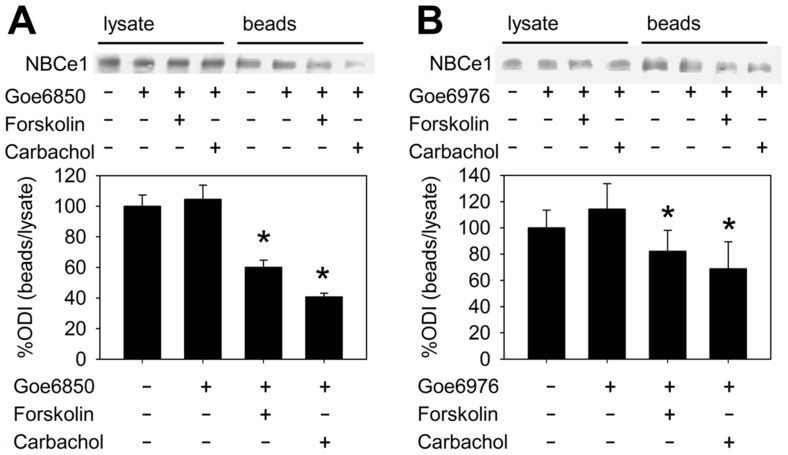
NBCe1 membrane expression during secretagogue stimulation and PKC inhibition with Gö6850 or Gö6976. In cell surface biotinylation experiments, neither Gö6850 (A; 5 μM) nor Gö6976 (B; 5 μM; continuous incubation for 20 min [+] vs. vehicle [−]) caused changes in NBCe1 surface expression. When forskolin (10^−5^ M) or carbachol (10^−4^ M) were added after 10 min, NBCe1 surface expression significantly decreased (40 and 59% for Gö6850, and 8 and 31% for Gö6976, respectively; n = 5–7 preparations from separate mice in each group, *:p<0.05, ANOVA for correlated samples followed by Tukey's HSD).

Since NBC is regulated by membrane trafficking [21], [24], [29], [30], and this mode of regulation has been reported to involve PKC signaling in the case of different basolateral membrane transport proteins [21], [31], [32], we analyzed NBCe1 membrane abundance in response to secretagogues. Having previously shown that forskolin and carbachol elicit their stimulatory effect on NBC at least partially via an increase in membrane expression [24], we investigated whether PKC is involved in secretagogue-induced NBC exocytosis. Preincubation with Gö6850 or Gö6976 alone did not alter NBC membrane expression, but caused a significant reduction of biotinylated NBC after stimulation with forskolin or carbachol (Figure 5).

## Discussion

The effect of PKC on Na^+^/HCO_3_^−^ cotransport is time-dependent and complex, and its investigation in various experimental systems has yielded controversial results. In the case of renal NBC, e. g., phorbol ester exerts a stimulatory effect in renal cells [22], [23], but inhibits PKC activity in hk(human kidney)NBCe1-transfected *Xenopus oocytes* [33]. Having previously reported PKC involvement in cholinergic NBC stimulation in native colonic tissue [8], we now sought to follow up on this observation, and set off to study the role of PKC in short-term modulation of NBC activity and membrane expression. Since NBC is mostly crypt-localized [2], we used isolated crypts for fluorometric and biotinylation experiments.

Given the stimulatory effect of secretagogues on colonic NBC, we first investigated PKC translocation, which was enhanced, and phosphorylation by these compounds. Interestingly, only the phosphorylation of PKC-α, but not of PKC-ε induced by secretagogues was sensitive to PKC inhibition. This may be explained by several published observations: The phosphorylation of PKC-ε appears to be more complex, with different PKC isoenzymes involved, and the phosphorylation state of conventional vs. novel PKC differentially contributes to its degradation probability [34], [35]. Furthermore, PKC-ε phosphorylation increases during mTORC1 activation after secretagogues and PMA, while PKC-α may even be degraded in response to these stimuli [36]–[39]. We believe that the differences between PKC-α and -ε regarding (auto-) phosphorylation-, degradation-, and regulation pathways contribute to the observed results. Our findings possibly point to a more prominent role of PKC-ε than PKC-α during secretagogue-dependent PKC stimulation. This would represent a parallel to the studies from the group of Jeffrey Matthews, who reported PKC-ε-dependent internalization of NKCC [40]. Together with our immunohistochemical data, we can conclude that PKC isoforms are present in the vicinity of NBC in the basolateral membrane, and are translocated and phosphorylated upon phorbol ester- and secretagogue exposure.

Having found that not only the known PKC activator carbachol, but also cAMP-dependent stimulation with forskolin can lead to PKC translocation and phosphorylation, we next fluorometrically assessed NBC activity after exposure to forskolin and/or PKC inhibitors. NBCe1-B is expressed at significant levels in murine colon [24], and although we cannot entirely exclude the contribution of other acid/base transporters, we have previously accumulated molecular and functional evidence that the Na^+^ and HCO_3_^−^ dependent, DMA-insensitive pH_i_ recovery we measure in crypts after imposing an acid load is mediated – at least predominantly – by NBCe1-B [2], [8]. Indeed, forskolin stimulation of NBC was reversed by PKC inhibition, which we had previously demonstrated for cholinergic stimulation [8]. While the group of Ira Kurtz had shown that the PKA-dependent phosphorylation site Thr^49^ is important for cAMP-dependent stimulation of NBCe1-B [10], these experiments indicate that not only cAMP-induced exocytosis represents an additional important component of NBCe1-B regulation [24], but that PKC is also a prerequisite for functional NBC activation elicited by forskolin. This adds to the complex and diverse regulatory roles of PKC, which mediates inhibitory effects on anion transport, such as the one of substance P on HCO_3_^−^ secretion in pancreatic ducts [17], but also stimulatory effects, such as phorbol ester stimulation of duodenal bicarbonate secretion in guinea pigs *in vivo* [18].

Given the importance of subcellular redistribution for NBC regulation [24], we hypothesized that membrane trafficking is part of the mechanism by which PKC inhibition reverses secretagogue stimulation of NBC. Apart from NBC, PKC is known to also modulate the membrane abundance of another major basolateral anion import mechanism in the intestine, namely Na^+^/K^+^/2Cl^−^-cotransporter [41], which represents the main import pathway for Cl^−^ destined for secretion. In the colonic epithelial cell line T84, phorbol ester down-regulates basolateral Na^+^/K^+^/2Cl^−^ cotransporter [42] by reducing NKCC1 surface expression via PKC-ε [40]. To evaluate this mode of regulation for NBC, we performed cell surface biotinylation experiments with carbachol-, forskolin- and PKC inhibitor-preincubation and found, unexpectedly, that PKC inhibition not only blocks secretagogue-induced exocytosis [24], but causes a significant reduction in NBC membrane abundance. Our experimental setup does not allow us to differentiate between membrane insertion and –retrieval, but this finding indicates that PKC is an elemental component of secretagogue-dependent NBC regulation, and that its inhibition may block the NBC membrane turnover to an extent that leads to decreased membrane expression. In the functional studies (Fig. 4), there was only a trend towards lower NBC activity, so these two measures do not fully quantitatively correlate. The reason may be that the decrease in NBCe1 membrane expression that occurs in the presence of PKC inhibitors despite secretagogue stimulation is in part compensated for by a functional activation of NBC itself by forskolin [2].

In two elegant studies, the group of Irina Gritchtchenko delineated cholinergic regulation of NBC subtypes transfected into a salivary gland cell line, and the role of PKC in this process [21], [30]. They observed an initial functional activation followed by NBCe1 endocytosis within the 5–15 minutes after stimulation, and interpreted this as a mechanism to adapt salivary secretion to a new steady-state [30]. In a follow-up study which separated the NBC and PKC isoforms/subtypes, they reported that PMA caused endocytosis of NBCe1-B, and that carbachol-dependent redistribution of NBCe1 into early endosomes is mediated by PKC-α, β, γ and novel PKC-ε [21]. Our approach using primary isolated crypts permits us to observe the primary events directly after stimulation, but due to their limited viability, we do not know whether the initial exocytosis and activity increase is also followed by redistribution to an intracellular compartment. Our results indicate that PKC is highly relevant for the stimulation-associated NBC exocytosis that leads to the activity increase after secretagogue exposure, with PKC inhibition causing a strong redistribution into an intracellular compartment.

One important question is whether other HCO_3_^−^ transporters are relevant. NBCn1, which is essential for duodenal acid-base-balance [3], [43], is also expressed in the colon [3]. The localization within the crypt-villus-axis, however, seems to be distinct, with NBCe1 being expressed in the upper part of the crypt, and NBCn1 in the lower part (unpublished). We have previously reported very low PCR expression levels for NBCn1 as compared with NBCe1 in isolated crypts [24], with the caveat that expression of different gene products is difficult to compare due to e. g. different primer efficiencies. In a more recent paper, NBCe1 and NBCn1 levels in the scraped colonic mucosa were comparable [3], which may be due to the fact that other structures such as blood vessels where NBCn1 has been described are present in this preparation. Despite the undisputable presence of NBCn1 in the lower colonic crypt, where it may serve a specialized function, there is thus substantial evidence indicating that NBCe1 is the predominant transporter when the entire crypt is taken into account. This was done in the present study by using isolated crypts for the biotinylation experiments, and regions of interest covering the bulk of the crypt in the fluorometric experiments. In a comparative expression study of SLC4 gene family members in human tissues, Damkier et al. reported a significant PCR signal for NBCe1, and a very low signal for NBCe2, NBCn1, and Cl-dependent NBC in the colon [44], the expression pattern overall being comparable to the existing data from rodents. Overall, the existing functional and molecular data gives us several clues that NBCe1 is important as a Na^+^/HCO_3_^−^ cotransporter in the murine colonic crypt, and there is no clear indication so far that the importance of other Na^+^/HCO_3_^−^ transporters comes close. However, we cannot entirely exclude a functional role of other Na^+^/HCO_3_^−^ transporters in our observations.

How exactly PKC exerts its regulatory effects on NBCe1 on a molecular level remains to be clarified. The question arises whether PKC acts exclusively via NBC endocytosis, or exerts an additional direct effect on the transporter. In salivary ParC5 cells, electrogenic NBC was also endocytosed by PMA [30], while this compound significantly reduced hk(human kidney) NBCe1 activity in *Xenopus oocytes*, without changing its membrane abundance [33]. Sequence comparison of the renal and the intestinal/pancreatic NBC subtypes revealed that the latter has a unique N-terminus of 85 amino acids, which replaces the first 41 amino acids in renal NBC [9]. This N-terminus contains 2 putative phosphorylation sites for protein kinase C, beginning at Ser^38^ and Ser^65^ [9]. As NBCe1-B possesses additional unique putative PKC phosphorylation sites [9], it is theoretically conceivable that PKC acts directly on NBC activity. Furthermore, PKC can inhibit the interaction of Slc26 family members and carbonic anhydrase (CA), a process termed “metabolon disruption” [45], and we have previously shown CA to be involved in cholinergic NBC stimulation [8], making this another potential regulatory mechanism for PKC. The recent discovery of an autoinhibitory module within the N-terminus of different NBCs, which is regulated by IRBIT and PIP2 [46] raises the question how PKC fits in and whether it acts sequentially or in parallel. Further studies involving site-directed mutagenesis modifying the previously identified putative PKC consensus phosphorylation sites unique to NBCe1-B [9] will have to solve this issue in the future.

## Supporting Information

Table S1 **Buffers used for the fluorometric experiments.** All buffers contained a combined buffering system including HEPES/TRIS. Osmolarity was 290–300 mOsm/l, and pH was adjusted to 7.4. TMA: tetramethylammonium, HEPES: (4-(2-hydroxyethyl)-1-piperazineethanesulfonic acid), TRIS: Tris(hydroxymethyl) –aminomethane.
(DOC)

## References

[1] Romero MF, Fulton CM, Boron WF (2004) The SLC4 family of HCO 3 - transporters. Pflugers Arch 447: 495–509. 14722772 10.1007/s00424-003-1180-2

[2] Bachmann O, Rossmann H, Berger UV, Colledge WH, Ratcliff R, et al (2003) cAMP-mediated regulation of murine intestinal/pancreatic Na+/HCO3- cotransporter subtype pNBC1. Am J Physiol Gastrointest Liver Physiol 284: G37–45. 12388213 10.1152/ajpgi.00209.2002

[3] Chen M, Praetorius J, Zheng W, Xiao F, Riederer B, et al.. (2012) The electroneutral Na+:HCO3- cotransporter NBCn1 is a major pHi regulator in murine duodenum. J Physiol. 10.1113/jphysiol.2011.226506 PMC3459045 22586225

[4] Jacob P, Christiani S, Rossmann H, Lamprecht G, Vieillard-Baron D, et al (2000) Role of Na+HCO3− Cotransporter NBC1, Na+/H+ Exchanger NHE1, and Carbonic Anhydrase in Rabbit Duodenal Bicarbonate Secretion. Gastroenterology 119: 406–419. 10930376 10.1053/gast.2000.9358

[5] Bachmann O, Franke K, Yu H, Riederer B, Li HC, et al (2008) cAMP-dependent and cholinergic regulation of the electrogenic intestinal/pancreatic Na+/HCO3- cotransporter pNBC1 in human embryonic kidney (HEK293) cells. BMC Cell Biol 9: 70. 19102757 10.1186/1471-2121-9-70 PMC2625339

[6] Ruiz OS, Arruda JA (1992) Regulation of the renal Na-HCO3 cotransporter by cAMP and Ca-dependent protein kinases. Am J Physiol 262: F560–565. 1314505 10.1152/ajprenal.1992.262.4.F560

[7] Ruiz OS, Qiu YY, Cardoso LR, Arruda JA (1997) Regulation of the renal Na-HCO3 cotransporter: VII. Mechanism of the cholinergic stimulation. Kidney Int 51: 1069–1077. 9083272 10.1038/ki.1997.149

[8] Bachmann O, Reichelt D, Tuo B, Manns MP, Seidler U (2006) Carbachol increases Na+-HCO3- cotransport activity in murine colonic crypts in a M3-, Ca2+/calmodulin-, and PKC-dependent manner. Am J Physiol Gastrointest Liver Physiol 291: G650–657. 16675744 10.1152/ajpgi.00376.2005

[9] Abuladze N, Lee I, Newman D, Hwang J, Boorer K, et al (1998) Molecular cloning, chromosomal localization, tissue distribution, and functional expression of the human pancreatic sodium bicarbonate cotransporter. J Biol Chem 273: 17689–17695. 9651366 10.1074/jbc.273.28.17689

[10] Gross E, Fedotoff O, Pushkin A, Abuladze N, Newman D, et al (2003) Phosphorylation-induced modulation of pNBC1 function: distinct roles for the amino- and carboxy-termini. J Physiol 549: 673–682. 12730338 10.1113/jphysiol.2003.042226 PMC2342979

[11] Gross E, Kurtz I (2002) Structural determinants and significance of regulation of electrogenic Na(+)−HCO(3)(−) cotransporter stoichiometry. Am J Physiol Renal Physiol 283: F876–887. 12372762 10.1152/ajprenal.00148.2002

[12] Glaser S, Alvaro D, Roskams T, Phinizy JL, Stoica G, et al (2003) Dopaminergic inhibition of secretin-stimulated choleresis by increased PKC-gamma expression and decrease of PKA activity. Am J Physiol Gastrointest Liver Physiol 284: G683–694. 12505882 10.1152/ajpgi.00302.2002

[13] Orsenigo MN, Tosco M, Baroni MD, Bazzini C, Laforenza U, et al (2002) Protein kinase C regulation of rat jejunal transport systems: mechanisms involved in bicarbonate absorption. Exp Physiol 87: 299–309. 12089597 10.1113/eph8702319

[14] Saksena S, Gill RK, Syed IA, Tyagi S, Alrefai WA, et al (2002) Inhibition of apical Cl-/OH- exchange activity in Caco-2 cells by phorbol esters is mediated by PKCepsilon. Am J Physiol Cell Physiol 283: C1492–1500. 12372810 10.1152/ajpcell.00473.2001

[15] Saksena S, Gill RK, Tyagi S, Alrefai WA, Sarwar Z, et al (2005) Involvement of c-Src and protein kinase C delta in the inhibition of Cl(-)/OH- exchange activity in Caco-2 cells by serotonin. J Biol Chem 280: 11859–11868. 15637072 10.1074/jbc.M411553200

[16] Tuo BG, Chow JY, Barrett KE, Isenberg JI (2004) Protein kinase C potentiates cAMP-stimulated mouse duodenal mucosal bicarbonate secretion in vitro. Am J Physiol Gastrointest Liver Physiol 286: G814–821. 14715523 10.1152/ajpgi.00251.2003

[17] Hegyi P, Rakonczay Z Jr, Tiszlavicz L, Varro A, Toth A, et al (2005) Protein kinase C mediates the inhibitory effect of substance P on HCO3- secretion from guinea pig pancreatic ducts. Am J Physiol Cell Physiol 288: C1030–1041. 15625303 10.1152/ajpcell.00430.2003

[18] Odes HS, Reimer R, Muallem R, Schwenk M, Beil W, et al (1996) Role of protein kinase C in duodenal mucosal bicarbonate secretion in the guinea pig. Pharmacology 53: 60–65. 8875602 10.1159/000139415

[19] Broughman JR, Sun L, Umar S, Scott J, Sellin JH, et al (2006) Chronic PKC-beta activation in HT-29 Cl.19a colonocytes prevents cAMP-mediated ion secretion by inhibiting apical membrane current generation. Am J Physiol Gastrointest Liver Physiol 291: G318–330. 16574993 10.1152/ajpgi.00355.2005

[20] Qu F, Liu HJ, Xiang Y, Tan YR, Liu C, et al (2011) Activation of CFTR trafficking and gating by vasoactive intestinal peptide in human bronchial epithelial cells. J Cell Biochem 112: 902–908. 21328463 10.1002/jcb.22999

[21] Perry C, Baker OJ, Reyland ME, Grichtchenko II (2009) PKC{alpha}{beta}{gamma}- and PKC{delta}-dependent endocytosis of NBCe1-A and NBCe1-B in salivary parotid acinar cells. Am J Physiol Cell Physiol 297: C1409–1423. 19783762 10.1152/ajpcell.00028.2009 PMC2793054

[22] Ruiz OS, Wang LJ, Qiu YY, Kear F, Bernardo A, et al (1996) Regulation of the renal Na-HCO3 cotransporter: VI. Mechanism of the stimulatory effect of protein kinase C. Kidney Int 49: 696–704. 8648910 10.1038/ki.1996.98

[23] Yamada H, Seki G, Taniguchi S, Uwatoko S, Nosaka K, et al (1996) Roles of Ca2+ and PKC in regulation of acid/base transport in isolated proximal tubules. Am J Physiol 271: F1068–1076. 8946002 10.1152/ajprenal.1996.271.5.F1068

[24] Yu H, Riederer B, Stieger N, Boron WF, Shull GE, et al (2009) Secretagogue stimulation enhances NBCe1 (electrogenic Na(+)/HCO(3)(−) cotransporter) surface expression in murine colonic crypts. AmJPhysiol GastrointestLiver Physiol 297: G1223–G1231. 10.1152/ajpgi.00157.2009 PMC3774290 19779011

[25] Espiritu DJ, Yang VL, Bernardo AA, Arruda JA (2004) Regulation of renal Na+/HCO3- cotransporter stimulation by CO2: role of phosphorylation, exocytosis and protein synthesis. J Membr Biol 199: 39–49. 15366422 10.1007/s00232-004-0675-x

[26] Schmitt BM, Biemesderfer D, Romero MF, Boulpaep EL, Boron WF (1999) Immunolocalization of the electrogenic Na+-HCO-3 cotransporter in mammalian and amphibian kidney. Am J Physiol 276: F27–38. 9887077 10.1152/ajprenal.1999.276.1.F27

[27] Hillesheim J, Riederer B, Tuo B, Chen M, Manns M, et al (2007) Down regulation of small intestinal ion transport in PDZK1- (CAP70/NHERF3) deficient mice. Pflugers Arch 454: 575–586. 17347851 10.1007/s00424-007-0239-x

[28] Martiny-Baron G, Kazanietz MG, Mischak H, Blumberg PM, Kochs G, et al (1993) Selective inhibition of protein kinase C isozymes by the indolocarbazole Go 6976. J Biol Chem 268: 9194–9197. 8486620

[29] Espiritu DJD, Yang VL, Bernardo AA, Arruda JAL (2004) Regulation of Renal Na+/HCO3− Cotransporter Stimulation by CO2: Role of Phosphorylation, Exocytosis and Protein Synthesis. Journal of Membrane Biology 199: 39–49. 15366422 10.1007/s00232-004-0675-x

[30] Perry C, Quissell D, Reyland M, Grichtchenko II (2008) Electrogenic NBCe1 (SLC4A4) but not electroneutral NBCn1 (SLC4A7) cotransporter undergoes cholinergic-stimulated endocytosis in salivary ParC5 cells. AmJPhysiol Cell Physiol. 10.1152/ajpcell.00153.2008 PMC2584990 18815229

[31] Song JC, Hrnjez BJ, Farokhzad OC, Matthews JB (1999) PKC-epsilon regulates basolateral endocytosis in human T84 intestinal epithelia: role of F-actin and MARCKS. AmJPhysiol 277: C1239–C1249. 10.1152/ajpcell.1999.277.6.C1239 10600776

[32] Liu J, Kesiry R, Periyasamy SM, Malhotra D, Xie Z, et al (2004) Ouabain induces endocytosis of plasmalemmal Na/K-ATPase in LLC-PK1 cells by a clathrin-dependent mechanism. Kidney Int 66: 227–241. 15200429 10.1111/j.1523-1755.2004.00723.x

[33] Perry C, Blaine J, Le H, Grichtchenko II (2006) PMA- and ANG II-induced PKC regulation of the renal Na+-HCO3- cotransporter (hkNBCe1). Am J Physiol Renal Physiol 290: F417–427. 16159892 10.1152/ajprenal.00395.2004

[34] Parekh DB, Ziegler W, Parker PJ (2000) Multiple pathways control protein kinase C phosphorylation. EMBO J 19: 496–503. 10675318 10.1093/emboj/19.4.496 PMC305587

[35] Durgan J, Cameron AJ, Saurin AT, Hanrahan S, Totty N, et al (2008) The identification and characterization of novel PKCepsilon phosphorylation sites provide evidence for functional cross-talk within the PKC superfamily. Biochem J 411: 319–331. 18237277 10.1042/bj20071348

[36] Carriere A, Romeo Y, Acosta-Jaquez HA, Moreau J, Bonneil E, et al (2011) ERK1/2 phosphorylate Raptor to promote Ras-dependent activation of mTOR complex 1 (mTORC1). J Biol Chem 286: 567–577. 21071439 10.1074/jbc.M110.159046 PMC3013016

[37] Leontieva OV, Black JD (2004) Identification of two distinct pathways of protein kinase Calpha down-regulation in intestinal epithelial cells. J Biol Chem 279: 5788–5801. 14638691 10.1074/jbc.M308375200

[38] Kim HW, Ha SH, Lee MN, Huston E, Kim DH, et al (2010) Cyclic AMP controls mTOR through regulation of the dynamic interaction between Rheb and phosphodiesterase 4D. Mol Cell Biol 30: 5406–5420. 20837708 10.1128/MCB.00217-10 PMC2976372

[39] Edelstein J, Hao T, Cao Q, Morales L, Rockwell P (2011) Crosstalk between VEGFR2 and muscarinic receptors regulates the mTOR pathway in serum starved SK-N-SH human neuroblastoma cells. Cell Signal 23: 239–248. 20851763 10.1016/j.cellsig.2010.09.008 PMC2956770

[40] Del Castillo IC, Fedor-Chaiken M, Song JC, Starlinger V, Yoo J, et al (2005) Dynamic regulation of Na(+)-K(+)-2Cl(−) cotransporter surface expression by PKC-{epsilon} in Cl(-)—secretory epithelia. Am J Physiol Cell Physiol 289: C1332–1342. 16000638 10.1152/ajpcell.00580.2004

[41] Tang J, Bouyer P, Mykoniatis A, Buschmann M, Matlin KS, et al (2010) Activated PKC{delta} and PKC{epsilon} inhibit epithelial chloride secretion response to cAMP via inducing internalization of the Na+-K+-2Cl− cotransporter NKCC1. J Biol Chem 285: 34072–34085. 20732874 10.1074/jbc.M110.137380 PMC2962506

[42] Matthews JB, Awtrey CS, Hecht G, Tally KJ, Thompson RS, et al (1993) Phorbol ester sequentially downregulates cAMP-regulated basolateral and apical Cl- transport pathways in T84 cells. Am J Physiol 265: C1109–1117. 8238301 10.1152/ajpcell.1993.265.4.C1109

[43] Singh AK, Xia W, Riederer B, Juric M, Li J, et al.. (2013) Essential role of the electroneutral Na+HCO3− cotransporter NBCn1 in murine duodenal acid/base balance and colonic mucus layer build-up in vivo. J Physiol. 10.1113/jphysiol.2012.247874 PMC3634528 23401617

[44] Damkier HH, Nielsen S, Praetorius J (2007) Molecular expression of SLC4-derived Na+-dependent anion transporters in selected human tissues. Am J Physiol Regul Integr Comp Physiol 293: R2136–2146. 17715183 10.1152/ajpregu.00356.2007

[45] Alvarez BV, Vilas GL, Casey JR (2005) Metabolon disruption: a mechanism that regulates bicarbonate transport. Embo Journal 24: 2499–2511. 15990874 10.1038/sj.emboj.7600736 PMC1176462

[46] Hong JH, Yang D, Shcheynikov N, Ohana E, Shin DM, et al (2013) Convergence of IRBIT, phosphatidylinositol (4,5) bisphosphate, and WNK/SPAK kinases in regulation of the Na+-HCO3− cotransporters family. Proc Natl Acad Sci U S A 110: 4105–4110. 23431199 10.1073/pnas.1221410110 PMC3593885

