# Supplementary material for: Short-Term Regulation of Murine Colonic NBCe1-B (Electrogenic Na+/HCO3 − Cotransporter) Membrane Expression and Activity by Protein Kinase C
Source: PLoS One. 2014 Mar 18;9(3):e92275. doi: 10.1371/journal.pone.0092275 (PMC3958514; doi:10.1371/journal.pone.0092275)
Supplement: Table S1 — Buffers used for the fluorometric experiments. All buffers contained a combined buffering system including HEPES/TRIS. Osmolarity was 290–300 mOsm/l, and pH was adjusted to 7.4. TMA: tetramethylammonium, HEPES: (4-(2-hydroxyethyl)-1-piperazineethanesulfonic acid), TRIS: Tris(hydroxymethyl) –aminomethane. (DOC) [file pone.0092275.s001.doc]

Table S1

|  | Buffer A | Buffer B | Buffer C | Buffer D |
| --- | --- | --- | --- | --- |
| NaCl | 120 | 100 | 60 | 0 |
| NaHCO_3_^-^ | 0 | 20 | 20 | 0 |
| Choline-HCO_3_^-^ | 0 | 0 | 0 | 20 |
| NH_4_Cl | 0 | 0 | 40 | 0 |
| TMA-Cl^-^ | 0 | 0 | 0 | 100 |
| HEPES | 14 | 14 | 14 | 14 |
| TRIS | 7 | 7 | 7 | 7 |
| KH_2_PO_4_ | 3 | 3 | 3 | 3 |
| K_2_HPO_4_ | 2 | 2 | 2 | 2 |
| MgSO_4_ | 1.2 | 1.2 | 1.2 | 1.2 |
| Ca^++^-gluconate | 1.2 | 1.2 | 1.2 | 1.2 |
| Glucose | 20 | 20 | 20 | 20 |
| gassed with | 100% O_2_ | 95% O_2_/5%CO_2_ | 95% O_2_/5%CO_2_ | 95%O_2_/5%CO_2_ |
